# Why is recruitment to trials difficult? An investigation into recruitment difficulties in an RCT of supported employment in patients with severe mental illness

**DOI:** 10.1016/j.cct.2008.07.007

**Published:** 2009-01

**Authors:** Louise Howard, Isabel de Salis, Zelda Tomlin, Graham Thornicroft, Jenny Donovan

**Affiliations:** a Health Service and Population Research Department, PO29 Institute of Psychiatry, King's College London, De Crespigny Park, London SE5 8AF, UK; b Department of Social Medicine and MRC Health Services Research Collaboration, University of Bristol, Bristol, UK

**Keywords:** Randomized controlled trials, Mental disorders, Qualitative research, Clinical trials as topic, Patient participation

## Abstract

**Background:**

Under-recruitment to randomised controlled trials (RCTs) is often problematic and there may be particular difficulties in recruiting patients with severe mental illness.

**Aim:**

To evaluate reasons for under-recruitment in an RCT of patients with severe mental illness

**Methods:**

Qualitative study during the recruitment phase of an RCT of supported employment. Trial staff and recruiting clinicians were interviewed. Data were analyzed thematically using constant comparative techniques.

**Results:**

Recruitment rates were low. Five main reasons for recruitment difficulties were found. These included: (i) misconceptions about trials, (ii) lack of equipoise, (iii) misunderstanding of the trial arms, (iv) variable interpretations of eligibility criteria, (v) paternalism.

**Conclusion:**

Reasons for recruitment difficulties in trials involving patients with severe mental illness include issues that occur in trials in general, but others are more specific to these patients. Clinician and patient involvement in the study design may improve recruitment in future similar trials.

## 1 Introduction

Recruiting patients to randomised controlled trials (RCTs) has long been recognized as a problem [1–3] with various strategies developed to overcome barriers to recruitment [4–7]. Recruitment delays can lead to increased costs accrued from funding extensions or to reduced power of the study [1,3]. Failure to recruit enough patients can result in early closure of the trial, unanswered scientific questions and wasted research resources. There may be particular difficulties in recruiting patients with severe mental illness into RCTs due to concerns about their potential vulnerability and reduced decision-making capacity [8]. However there is a lack of published research into whether patients with severe mental illness are difficult to recruit and why this may occur. It is clearly important that this group is not excluded from research. The aim of this paper was to evaluate reasons for under-recruitment in an RCT of patients with severe mental illness.

### 1.1 Method

This qualitative study was carried out during the recruitment phase of the SWAN (Supported Work and Needs) trial. The SWAN trial is an RCT of supported employment provided by employment consultants integrated within community mental health teams (CMHTs) with the aim of focusing on rapid placement into jobs and continued follow-up support. This intervention was compared with usual care which consisted of existing psychosocial rehabilitation programmes available in the local area. Unlike the intervention arm, the services in the control group were coordinated with, but not integrated into, the CMHTs.

Recruitment involved a two stage process. First, the care coordinators (CCs) (mental healthcare professionals with a background in nursing, social work or occupational therapy who coordinate patient care and provide face to face care) in CMHTs were asked to identify patients who fulfilled the inclusion criteria for the SWAN trial (psychotic or chronic affective disorder for over 2 years, age 18–65). The CCs explained the trial to these patients and asked their permission to be contacted by a researcher. The researcher wrote to patients inviting them to an appointment to further discuss the trial. Enclosed with this invitation letter was a patient information sheet. After meeting the potential participant and explaining the trial, the research nurse (R) or trial coordinator (TC) contacted the central randomization service to arrange randomization for those patients who gave consent to enter the trial. The trial coordinator and research nurse were therefore responsible for liaison with CMHTs, discussing potential subjects with CCs and recruiting patients after they had been approached by CCs.

All staff of participating CMHTs were invited to attend presentations from trial staff explaining the SWAN trial. These presentations took place at the team base (community mental health centres) to maximise attendance. Information on the trial was also provided in regular newsletters, advertisements and printed summaries of the protocol. This included details of local and national vocational agencies available for patients randomised to the control arm. The recruitment period started November 2004 and was originally planned to last 18 months. However, this was extended to 2 years because of slow recruitment. The Quartet team was invited to explore reasons for slow recruitment after the first year of the trial when 100 patients had entered the trial (the recruitment target was 216 patients so the planned total after 1 year was 144).

## 2 Quartet methods

The Quartet (Qualitative research to improve recruitment to randomised controlled trials) study works through collaboration with trial management groups to investigate whether qualitative research methods can be used to improve recruitment to RCTs experiencing or anticipating poor recruitment. The Quartet study focuses on understanding how the trial is presented to potential participants throughout the recruitment process, providing evidence about recruitment difficulties, and then, if possible, the development of an intervention to improve recruitment, often involving training or feedback to recruitment staff. The research methods were developed from the ProtecT (Prostate testing for cancer and Treatment) feasibility study [9] which successfully increased randomization rates. Methods include interviewing trial staff, either in groups or individually, and recording and analyzing the eligibility and recruitment appointments between health professionals and patients. These methods are adapted for individual trials to unearth barriers to recruitment. Data from interviews, focus groups and appointments are transcribed and analysed using techniques of constant comparison to identify important themes.

## 3 Collaboration with SWAN: data collection and research methods

In collaboration with the SWAN trial, the Quartet team decided to focus on interviews with trial and clinical staff and not audio-recordings of recruitment appointments. This was for two reasons. Firstly, the key recruitment stage was not the recruitment appointment with the researcher — by which time most patients had already decided to join the trial — but when CCs first introduced the trial to patients to see if they agreed to be contacted by the researcher. This crucial recruitment stage was difficult to capture by audio-recording as it occurred informally at any time and place as part of CCs' clinical work. Secondly, the recruitment period did not allow enough time for audio-recording and feedback.

The Quartet team initially interviewed four trial staff — Chief Investigator (CI), Principal Investigator (PI), trial coordinator (TC) and the research nurse (R) — followed by two CCs, one of whom was a team manager. Interviews broadly covered the recruitment process, perceived difficulties with the trial and how it was run. They also focused on the acceptability of trial arms, understanding of randomization and how eligibility was decided. After the interviews were transcribed, themes arising from the data were coded using ATLAS software. Codes were compared across the data set and cross checked by two independent researchers to ensure inconsistencies were accounted for and to agree on significant themes [10]. The findings were then fed back to trial staff.

Following consultation with trial staff, the Quartet team then led two workshops with two different teams of CCs, based on the findings of the qualitative research. The original aim of the workshops was for them to be an intervention to feed back CCs' difficulties with the trial and to challenge misconceptions and misunderstandings. However, practical barriers, including governance procedures and reorganization of teams delayed this, so that the workshops occurred near the end of the recruitment period and their impact on recruitment could not be evaluated.

The key issue of eligibility was explored in the workshops through discussing four hypothetical scenarios of patients who were considered eligible for the trial by the PI. The aim of these discussions was to ascertain whether CCs would refer these patients as potential participants and whether there was consensus on this. Two teams of CCs (out of 10) were invited to participate in the workshops, based on their expressed willingness and convenient locations. Out of these two teams, seven CCs attended the first workshop and ten attended the second. Although only a relatively small number of CCs and teams were represented, others were included in the study through interviews, and the findings were consistent. The workshops were organized so that trial staff joined each group halfway through the session, allowing CCs initial freedom to discuss the trial and later to ask questions of trial staff.

## 4 Results

The aim throughout the collaboration was to understand the reason for the low level of recruitment. Five key themes emerged from the interviews and workshops reflecting views about recruitment, and each is described in detail below.

### 4.1 Misconceptions about RCTs

CCs, who were very important in initially approaching patients and introducing the study, held misconceptions about randomised controlled trials (RCTs) despite being familiar with the terms “trial” or “RCT”. Many CCs did not realise SWAN was a study that randomly allocated patients to either the control or intervention, and some had difficulty with the role of randomization (Table 1). The CC in extract 1 had not understood his patients only had 50% chance of getting the intervention, despite knowing it was a trial. In the second extract, the CC did not realise that the trial had two arms, that her patients had received the control arm, and that the process of randomization determined which arm a patient received. In the third extract, a CC expressed difficulty in explaining randomization to patients. In the fourth extract the CC did not understand that getting the control arm was the product of random selection, but saw it as rejection from the trial.

These comments also reflected that patients and CCs equated the intervention with the trial as a whole, a misconception arising out of the trial name (Supported Work And Needs — SWAN), so that the whole SWAN trial was equated with the intervention. This confusion was apparent in the way CCs described their patients' feelings when receiving the control arm. As exemplified in Table 1 (extracts 3, 4 and 5), not being included in the intervention arm was commonly described in strong terms as “rejection” or “a massive blow”. The experience of rejection expressed by CCs and their patients went beyond feeling they had somehow failed to be selected. They seemed to feel that patients had been rejected despite needing and being suitable for work (extract 5). The sixth extract reiterates these points that the patient did not understand they were part of a trial and that the trial was being confused with the intervention. This conflation was possibly exacerbated by the lengthy baseline interview (1–11/2 h) between the researcher and patient before randomization.

Trial staff worked hard at communicating with the health workers they relied on for the trial to work. There was regular contact with the researcher, repeated presentations to teams and repeated explanations of the trial (extract 7). A list of alternative vocational agencies for the control arm was provided at the team presentations and enclosed in letters to CCs whenever a patient was randomised to the control arm. However, the CCs clearly still had misunderstandings about the trial, could not ground it in their daily practice or make sense of it to themselves or their clients. This was not helped by the restructuring of the teams which led to changes in who was working within the CMHTs.

### 4.2 Equipoise

Expressing the control arm as rejection not only reflected misconceptions about the trial but also a lack of equipoise between the two arms. CCs did not accept that the control arm was in any way equivalent to the intervention arm, overwhelmingly favouring the intervention (Table 2). As CCs assumed supported employment would be better for their patients than usual care (extract 1), they were often unsupportive of SWAN, considering the intervention should be part of normal practice and not be tested in an RCT (extract 2). One CC exemplified this attitude by describing the intervention as a specialist service with the potential to provide more than they themselves could offer (extract 3).

It was not only CCs who favoured the intervention arm over the control arm; trial staff also expressed difficulty in maintaining equipoise. Both the research nurse and PI explained they considered the intervention arm to be more successful in helping patients get work (extracts 4 and 5). Not surprisingly, some patients randomised to the control arm who were disappointed with their allocation sought to obtain the active intervention.

### 4.3 Misunderstanding the trial arms

A key problem for the trial was that CCs did not understand the control arm of usual care and lacked information about how to present it. Although trial staff referred to a list of possible alternative employment agencies to which CCs could refer clients, some CCs had no knowledge of this list (extract 1, Table 3). The Chief Investigator (CI) considered that a major problem with the control arm was that the notion of what constituted ‘usual care’ was changing. Whereas CCs may previously have not considered proper employment for their patients, they now did, in part because of the trial (extract 2, Table 3).

### 4.4 Eligibility

It became apparent in interviews that CCs had idiosyncratic interpretations of who was eligible for the trial. Furthermore, the way a patient's eligibly status was decided varied from team to team. Whereas some CCs judged patients individually, others reached consensus during team meetings. The approach of one team was to use a zoning system which considerably reduced the pool of eligible patients (extract 1, Table 4).

There was widespread fear among CCs that the stress of inappropriate employment could trigger patients to relapse. An exercise in the workshops was to discuss four potential case scenarios, deemed eligible for the trial by the PI, and explore whether CCs would refer them to the researcher. In the ensuing debates several CCs stated that they would not refer these patients to SWAN, a common reason being that the stress of work would lead to their patients relapsing. However, there was not necessarily consensus on this as shown in the second and third extract. CCs used their own perceptions of who was ready to work, not necessarily in accordance with each other. This varied from being under motivated due to low self-esteem or over motivated if not yet “ready” for employment (extract 4). Some CCs defined patients who had chaotic lifestyles and could not take on responsibility as unable to manage the intervention, giving the impression that they made these decisions on behalf of their patient (extract 5,). Finally, many teams were being re-organised and taking over the care of unknown patients. CCs felt that they could not discuss work with patients until they had a clearer understanding of patients' needs irrespective of whether or not they fitted into the trial eligibility criteria (extract 6).

### 4.5 Paternalism: conflict in roles

Both the issues of eligibility and equipoise reflected the conflict between being a researcher recruiting for a trial and a health professional protecting the interests of patients. Many of the above extracts reveal that CCs primarily thought of themselves as their patients' carers, focusing more on their perception of patient needs than providing patients with the opportunity to decide whether they would like to participate in the research. As indicated above, CCs felt patients should be protected from the stress of employment. Some patients were also perceived as potentially too psychologically fragile to deal with the consequences of being “rejected”, or allocated to the control arm (extract 1, Table 5). The trial team described the relationship between CCs and patients, where the health professional decides on their patient's suitability for work, as paternalistic. CCs acknowledged that they were a bit “overprotective” of their patients (extract 2, Table 5).

### 4.6 Subsequent recruitment

As a result of these study findings, we subsequently provided additional written and verbal information to teams, and we were successful in exceeding our recruitment target by the end of the second year (recruiting 219 patients to the trial). However as the study was carried out relatively late in the recruitment phase (partly because of delays in receiving the relevant institutional approvals) it was not possible to assess the impact of the intervention systematically.

## 5 Discussion

This study found five main reasons for recruitment difficulties in this RCT. First, care coordinators (CCs), the clinicians who initially approached patients and introduced the study, did not appear to understand the concept of an RCT. Second, they had difficulty in expressing equipoise. Third, research and clinical staff had difficulty in accepting the usual care arm, and patients did not always understand the difference between participation in the RCT and receiving routine care, known as the “therapeutic misconception”. Fourth, CCs applied their own eligibility criteria, not necessarily in accordance with each other. Finally, they appeared paternalistic and focused more on their perception of patient needs, such as avoiding the stress of looking for a job or the psychological consequences of the disappointment of being randomised to the control arm, than providing patients with the opportunity to decide whether they would like to participate in the research. These misconceptions existed despite trial staff's efforts to provide on-going support and address misunderstandings.

Some of these recruitment issues are known features of RCTs in general whilst others are more specific to this particular trial. It is known that clinicians involved in recruiting to RCTs can have difficulties providing information on key aspects of the research process, for example randomization and equipoise [9,11–13]. This difficulty was exacerbated in this trial by the fact that recruiting clinicians had little interest in research and equated the intervention with the trial. Lack of eligible patients is a major theme for many trials [14] with clinicians employing their own interpretations of the eligibility criteria [15,16]. Though it is understandable that clinicians may want to interpret eligibility criteria according to their own clinical experience, this does have important implications for a trial if this differs from the trial protocol and reduces and skews the pool of eligible patients. Such practices can have a negative impact on recruitment and affect the generalisability of results to a clinical population [18]. In this trial the interpretation of who was eligible was underpinned by the paternalistic role that care managers adopted towards their patients.

A clear finding was the lack of individual equipoise — defined as genuine uncertainty about which trial arm is better [17]. Usual care (treatment as usual — TAU) was perceived by trial staff and patients as substandard in comparison to the intervention arm; other researchers have also reported that if a trial arm is perceived as less desirable this can cause recruitment problems [18]. TAU as a control arm is generally considered to be a reasonable and ethically acceptable standard against which to measure the effectiveness of a new treatment, and the bedrock of pragmatic RCTs. In this trial TAU was provided by vocational services delivered by providers outside the direct influence of the trial. Care coordinators were confused about the nature of TAU and could not properly describe it to their patients. This was for two reasons. They considered the TAU arm was effectively ‘no care’. In part this was because the trial had sensitized them to the idea that competitive employment could be beneficial for their patients; consequently vocational agencies that did not focus on competitive employment for people with severe mental illness were not considered as good. Second, they appeared confused about what actually constituted TAU, partly because this did not consist of a single understandable entity that provided similar services everywhere.

If participants do not understand the purpose of a trial, they may believe that the purpose of the trial discussion is to provide the best individual care for them. This is known as the therapeutic misconception. This can result in underestimating the risks or overestimating the benefits of research participation and thus hinder informed decision-making [19]. In this trial patients and their clinicians felt let down when they received the TAU arm, because they felt it implied ‘no care’. A number of authors have discussed the relevance of the therapeutic misconception to the ethics of schizophrenia research [19–21] but this phenomenon has also been described in many non-psychiatric research populations (e.g.[22–26]). Researchers have found that in patients with schizophrenia or schizoaffective disorder, less education or worse cognitive functioning is associated with higher levels of therapeutic misconception [19]. This means that it would be helpful that understanding is not assumed but checked when working with this patient group.

Conflict in the roles of researcher and clinician is a recognised problem for health professionals recruiting to trials [3,15,27,28]. What is specific to this trial is the extent of paternalism found between the clinicians and their patients, reflecting the fact that the patient group are patients with chronic mental illness and so perceived as especially vulnerable. This has particular consequences for recruiting to mental health trials. The recruiter–patient relationship is typically long-term, with clinicians acting partly as their patients' advocates. Although no one knows before a relapse how much ‘stress’ will be bearable, the goal in such a therapeutic context was often to avoid new challenges including those in this trial: work or participating in research. CCs were also not part of a research culture. The level of paternalism here meant that some, perhaps many, individuals were being denied access to the trial and not allowed to make an informed choice about participating in the research.

These findings are based on a small sample of interviewees, which is a major limitation of this study. Information was only disseminated directly to CCs who attended the workshops, so any impact on recruitment processes would be limited as non-attenders would not have received important information. However, we subsequently also changed written information to the CCs which was sent to all team members. In addition, as with all retrospective studies, there is the potential for recall bias, particularly as care coordinators and researchers may have been looking for reasons for difficulties in recruitment during interviews which took place in the context of poor levels of recruitment. This study is also specifically related to recruitment strategies that rely on clinicians' indication, and other trials that do not rely on clinicians (e.g. media advertisements, specific screening) are likely to be associated with different recruitment issues.

Nevertheless, these findings may be relevant to trials with similar recruitment procedures and highlight the need for further research in this area. The findings also have practical implications for future trials recruiting patients with severe mental illness. Concern among research and clinical staff about the vulnerability of research participants should be acknowledged and discussed from the outset. Some researchers have reported that impaired decision-making capacity can be addressed by specific interventions [29–31] and such interventions should be considered, particularly in patients with cognitive impairment as this is the main predictor of incapacity in this patient group. Recruiting clinicians also need to be very clear on the reasons for the research, the eligibility criteria, the role of randomization, the process of the study and the nature of the control arm. Usual care as the comparison needs to be carefully explained and understood by those recruiting and participating in the trial. These findings show that the Quartet methods of listening to recruiters are essential in order to understand the issues from their perspective. Presenting to and talking to recruiters may not be enough.

It is perhaps understandable that recruitment is especially difficult when the process relies on clinicians who know little about trials and do not have a career interest in research. For this reason, clinician and patient involvement in study design may prove vital in improving recruitment and participation in similar RCTs. It is clear in retrospect that if trial staff had initially elicited and explored CCs' views about the rationale for the trial and proposed recruitment practices they could have determined the acceptability of these to those recruiting and their patients. Trial staff need to recognize that health professionals may feel ambivalent about research, especially if it appears of no benefit to themselves or their patients; addressing this may help trialists overcome some recruitment hurdles in the future.

## Figures and Tables

**Table 1:** Misconceptions about RCTs

*Extract 1*
CC2: SWAN is still a study isn't it and it's still only fifty fifty and there was a little bit of misrepresentation when they first came out, because basically fifty percent of the guys were being told right no we can't do anything for you, whereas you'd sort of let us, they let us sort of think that it was a sort of grounded, established service to provide employment help… they said it was a trial this that and the other but they certainly didn't say that you know there's a good chance that your clients aren't going to get (the supported employment). (Interview)
*Extract 2*
CC4: I referred three of my clients to these at the SWAN and I just got a letter back saying that none of them had been taken on, but I didn't know why.
QR (Quartet researcher): You didn't know why, Ohh.
CC4: No I just got this letter back saying, “No, we've not taken them.” But I would have liked to have known actually why.
QR: But do you understand now, why? From what I've said, that they were randomised?
CC4: Yeah okay
QR: And the computer programmes, just by chance drew out the control arm for them?
CC4: Yeah, okay (WS1)
*Extract 3*
CC7: With these random control trials, I had some clients, they were keen but they don't know why they are getting put on the computer and were not picked and then people that were not keen were being picked. So what am I meant to do? (WS2)
*Extract 4*
CC8: It depends if she's robust enough to take the rejection if she doesn't get into the trial I suppose. I suppose you would have to explain that to her that she may not get in.
TS: What do you mean by get into the trial?
CC10: Well, into the intervention group I think she means. (WS1)
*Extract 5*
CC2: But then … it was really important that this guy got the support helping him back to work that he needed. He got rejected, and it was a massive blow, … really hit him hard ‘cos he wasn't, I don't think he was expecting it either. (Interview)
*Extract 6*
CC5: I've got some written feedback from the client that went to SWAN, which isn't positive because she felt that she told them everything about herself, which was quite — she felt was quite personal and then she was told that she wasn't given the service…. So it's sort of — she felt that she went through quite a sort of process only to be said — told that no, you don't get the service. (WS1)
*Extract 7*
R: I'd been explaining what randomisation was for the last year, and nobody had understood it. And there's a temptation to think well, there's something about me that's not explaining this right. And you know in fact, to have three other people explaining it, (trial supervisor) had explained it, and you had explained it, (Quartet member) had explained it, and still some of the same questions were coming back (final interview).

**Table 2:** Lack of equipoise

*Extract 1*
CC7: The intervention arm is better and the reason why I believe is that it provides the extra support that the clients require to pass through the selection and interview and help in gaining a job. Also the clients believe that, apart from their employer, they have somebody that they can seek support from because the SWAN shall make them aware if they have problems. And also the third one (reason) which I am about to (say) is that the employer also has prior knowledge about the individual, that if they are relapsing they can inform the SWAN. (WS2)
*Extract 2*
CC2: I think basically you either get support or you don't. That's the way, that's what it seems like at ground level
QR: Yeah, and support is a good thing?
CC2: Oh yeah
QR: Yeah, and do you think the trial's justified then?
CC2: No I don't think it's justified, it should be a service you know?
QR: Straightforward service?
CC2: Absolutely (Interview)
*Extract 3*
QR: Okay, do you ever have a personal feeling about which arm the patient will do better in, when you actually get to see the patient?
R: Absolutely, yeah, I always want the intervention group (for them). And I feel like I hate telling them they're in the control group, I absolutely hate it…But yeah definitely I always will want the intervention group.
QR: Why is that?
R: I suppose because if they're expressing a need for work and they want work and they want help in looking for a job then I want them to get that help. (R: Interview)
*Extract 4*
QR: Your view of supported employment right at the beginning of the trial?
PI: Right at the beginning I thought it would probably help.
QR: So you were not in equipoise you thought that-?
PI: I was not in equipoise, but that was supported employment, the American model. But it became increasingly clear that we weren't actually testing the American model, so I had more equipoise as the study went on, I would say yep. (TS: Final Interview)

**Table 3:** Misunderstanding the trial arms

*Extract 1*
CC4: They were allocated out (control arm). I haven't done anything
QR: So you didn't refer them to another service then?
CC4: No…
QR: Because the idea behind the trial is that if your client gets allocated to the control arm then they're not getting supported employment, and then there is this list apparently of other options that you can use for them if they feel that they still are interested in going for some kind of employment— for jobs — and you didn't you know that?
CC5: No
CC6: No (lots of laughter), but we know now (laughs).
CC5: I mean we haven't discussed it and we didn't know that we had access to these resources (WS1)
*Extract 2*
CI: Until the last year or so, the question of real open market paid employment for people with more disabling conditions rarely came into the picture for clinical teams…Not only was it not really often discussed or any action taken, but rarely did the staff actually refer patients to this particular employment agency. Which they could have done, but they didn't. So it wasn't as if this was a sort of widely used option and it's a valuable resource and many people going that way. …I think it's two things, one is the trial sensitised staff to the question of work, but also that the wider health policy environment has changed. (Interview)

**Table 4:** Interpreting eligibility

*Extract 1*
CC1: We have three zones, it's like the traffic light system. Red obviously is for clients who we're really concerned about who's not very well. Amber's for clients who are fairly stable and ticking over quite well and the green zone is for clients who have been maintaining wellness for quite sometime who we feel would be able to move back into the community with support, but also having the backup there just in case something was to go wrong. There are a number of these clients who we refer on to SWAN, clients who we feel are quite stable and would benefit from a structure,… full time activity and preparing them for employment… These are the green clients…the amber ones we tend to wait until they're in the green bank…. Never the clients in the red zone ‘cos obviously they're mentally not that well at the moment, at that time, at that stage. (Interview)
*Extract 2*
CC7: I wouldn't refer them. I wouldn't refer them.
QR: Really, why?
CC11: I wouldn't.
CC12: I wouldn't.
QR: Why?
CC7: …So with someone like this you get them a job and within two or three months they are relapsed, back in the hospital, the pressure is too much, it's like they are not really well enough, because three months they are still having a good and bad day, they are still complaining about their current medication, they need to keep taking their medication.
CC3: But for me maybe the bad days are because she hasn't got anything to do, nothing meaningful to do during the day. I would be more likely to refer this lady because she is motivated, she is saying that she wants to go out…so you have to take that on, she would like to go out to work. (WS 2)
*Extract 3*
CC8: I mean that all of our work is trying to sort of minimise (trigger factors) and any change is going to enhance someone's stress and vulnerability aren't they? So even trying something new even if it is positive, if she doesn't get on it, it may increase her stress and vulnerability and she may start abusing (drugs) again, so I suppose that would be your concern isn't it? I think if things are stable, let's leave them as they are. Let's not add something into the mix that might make her stressed again which might make her (relapse) (WS1)
*Extract 4*
CC1: and a lot of people too, we've found is that they've not worked for a very long time so the confidence is not there, the self-esteem is quite low, the motivation is not there at all so it's working on those self-esteem issues and motivation issues and to get them to the point where they feel that they would want to take that chance of going back out into mainstream and finding some work, and there are some who feel overly ready but you know yourself that they're not ready, it's those…that's where it's quite difficult to work it. (Interview)
*Extract 5*
QR: And are there certain people you definitely wouldn't (refer)?
CC2: Oh absolutely, I mean yeah another one of my guys, although this one in particular is a very gifted craftsman, but you know his lifestyle, drugs and chaotic lifestyle is just totally inappropriate to meet any sort of commitments or take any responsibility even to getting up and you know, and attending when you should, yeah ‘cos there's quite a few of our guys who are too chaotic. (Interview)
*Extract 6*
CC1: For me definitely is that we don't know a lot of the clients that we've recently inherited. So for us to be able to make referrals we need to get a better understanding about the clients needs and I don't feel that we've reached that stage yet. (Interview)

**Table 5:** Paternalism: conflict in roles

*Extract 1*
CC8: So say people come with very complex issues about rejection and you know this may feel like another rejection to them and we may have as clinicians, we may feel we don't want to put our clients through that. (WS1)
*Extract 2*
CC3: I think that maybe we are a bit overprotective of the client group that we work with because the majority of them have been through the system and we tend to be at the latter end of the system, where we are trying to put them into a situation where they feel good about themselves about going back to work…
CC9: Plus we have a therapeutic role with the client and we are acutely aware that the NHS is a huge and sort of mind boggling organisation for a lot of the clients and they don't want to feel let down by the mental health system and it's so easy for them to feel that way. (WS2)
